# Regulation of metabolism in *Escherichia coli* during growth on mixtures of the non-glucose sugars: arabinose, lactose, and xylose

**DOI:** 10.1038/s41598-017-18704-0

**Published:** 2018-01-12

**Authors:** Ehab M. Ammar, Xiaoyi Wang, Christopher V. Rao

**Affiliations:** 1 Department of Chemical and Biomolecular Engineering, University of Illinois at Urbana-Champaign, Urbana, IL 61801 USA; 2 Genetic Engineering and Biotechnology Research Institute, University of Sadat City, El-Sadat City, Egypt

## Abstract

Catabolite repression refers to the process where the metabolism of one sugar represses the genes involved in metabolizing another sugar. While glucose provides the canonical example, many other sugars are also known to induce catabolite repression. However, less is known about the mechanism for catabolite repression by these non-glucose sugars. In this work, we investigated the mechanism of catabolite repression in the bacterium *Escherichia coli* during growth on lactose, L-arabinose, and D-xylose. The metabolism of these sugars is regulated in a hierarchical manner, where lactose is the preferred sugar, followed by L-arabinose, and then D-xylose. Previously, the preferential utilization of L-arabinose over D-xylose was found to result from transcriptional crosstalk. However, others have proposed that cAMP governs the hierarchical regulation of many non-glucose sugars. We investigated whether lactose-induced repression of L-arabinose and D-xylose gene expression is due to transcriptional crosstalk or cAMP. Our results demonstrate that it is due to cAMP and not transcriptional crosstalk. In addition, we found that repression is reciprocal, where both L-arabinose and D-xylose also repress the lactose gene expression, albeit to a lesser extent and also through a mechanism involving cAMP. Collectively, the results further our understanding of metabolism during growth on multiple sugars.

## Introduction

During growth on a mixture of sugars, bacteria will often consume the sugars sequentially through a process known as catabolite repression^1,2^. Usually, the cells first consume the sugar yielding the highest growth rate, followed by the sugar yielding the next highest growth rate, and so on^3^. They do this by repressing the expression of the genes involved in metabolizing the less preferred sugars^2^. This mechanism maximizes the growth rate by ensuring that cells devote their limited metabolic resources towards the preferred sugars^2^. The classic example involves the growth of the bacterium *Escherichia coli* on glucose and lactose, where *E. coli* will consume glucose before lactose^1,4,5^. The sequential utilization of these two sugars results in diauxic growth, where the cells first grow on glucose and then, following a short period of no growth, proceed to grow on lactose. This two-phase pattern of growth is also observed when *E. coli* is grown on mixtures of glucose and some other sugars^2^.

In *E. coli*, glucose catabolite repression is regulated by the phosphotransferase system, a multi-protein phosphorylation cascade that couples glucose uptake and metabolism^4,6,7^. One protein in this cascade, EIIA^Glc^, plays a central role in glucose catabolite repression. When EIIA^Glc^ is phosphorylated, it activates adenylate cyclase, which increases the concentration of cAMP in the cells. cAMP activates the expression of many genes involved in the metabolism of non-glucose sugars through the action of the cAMP receptor protein CRP. In addition, the expression of these genes is also induced by their cognate sugars. Because EIIA^Glc^ phosphorylation increases when the cells are not metabolizing glucose, these CRP-regulated metabolic genes are expressed only in the absence of glucose and presence of their cognate sugar. In addition, unphosphorylated EIIA^Glc^ can bind and inhibit the ability of some sugar transporters to uptake their cognate sugars through a process known as inducer exclusion^4,6–10^. Thus, when the cells are metabolizing glucose and EIIA^Glc^ is predominantly in the unphosphorylated form, these sugars are not transported into the cell and, therefore, are unable to induce the expression of their cognate metabolic genes. These two mechanisms are believed to be the main factors regulating glucose catabolite repression in *E. coli*.

Catabolite repression has also been observed with sugar mixtures not involving glucose. One prominent example is the growth of *E. coli* on mixtures of L-arabinose and D-xylose (hereafter referred to simply as arabinose and xylose)^11^. In this case, the cells will consume arabinose before xylose, though the extent of repression is less pronounced than observed with glucose and arabinose or glucose and xylose. Arabinose catabolite repression results from regulatory crosstalk between these two sugar utilization systems^12^. The arabinose transport and metabolic genes are regulated by the transcription factors AraC and CRP. When AraC is bound with arabinose and CRP with cAMP, they activate the expression of the arabinose genes. Similarly, the xylose transport and metabolic genes are regulated by the transcription factors XylR and CRP. When XylR is bound with xylose and CRP with cAMP, they activate the expression of the xylose transport and metabolic genes. Arabinose catabolite repression results from arabinose-bound AraC binding to the promoters of the xylose genes and inhibiting their expression through a competitive mechanism. In addition, xylose weakly represses the expression of the arabinose genes using an analogous mechanism involving xylose-bound XylR. This reciprocal mechanism generates a small subpopulation of the cells that selectively consume xylose even in the presence of arabinose^13^.

A recent study by Aidelberg and coworkers investigated the utilization of six non-glucose sugars (lactose, arabinose, xylose, D-sorbitol, L-rhamnose and D-ribose) in *E. coli*^14^. They observed hierarchical expression of the metabolic genes for these sugars. In particular, they found that sugars higher up in the hierarchy repress the expression of the genes involved in the metabolism of the sugars lower down in the hierarchy. These results suggest that the cells selectively utilize these six sugars in a similar hierarchical manner. In addition, they proposed that the hierarchical expression of these genes is regulated by CRP through the differential production of cAMP. A number of studies have shown that cAMP levels are inversely proportional to the growth rate of the cells, irrespective of the sugar, due to inhibition of adenylate cyclase by the downstream metabolites^15–17^. In support of this hypothesis, Aidelberg and coworkers found that the expression hierarchy matches the growth rates associated with these sugars. Furthermore, they found that the promoters for genes associated with sugars at the top of the hierarchy are more sensitive to cAMP than those at the bottom of the hierarchy. In other words, when the cell is growing on a higher-growth yielding sugar such as lactose, insufficient cAMP is produced to induce the expression of genes for lower-growth yielding sugars such as rhamnose. This mechanism is appealing as it readily explains how cells selectively utilize sugars in accords with their growth yield. These results also suggest that multiple mechanisms are involved in the selective utilization of non-glucose sugars, where some involve transcriptional crosstalk, as is the case with arabinose and xylose, and others do not.

In this work, we investigated the selective utilization of lactose, arabinose, and xylose. When *E. coli* is grown on a mixture of lactose and arabinose or lactose and xylose, it will consume the lactose first. Motivated by the study of Aidelberg and coworkers, we sought to determine whether the preferential utilization of lactose is due to cAMP or regulatory crosstalk. Using a number of different approaches, we conclude that the selective utilization of lactose is mainly due to cAMP. These results indicate that *E. coli* employs multiple mechanisms to regulate the utilization of non-glucose sugars.

## Results

### Lactose represses arabinose and xylose gene expression

**Figure 1:**
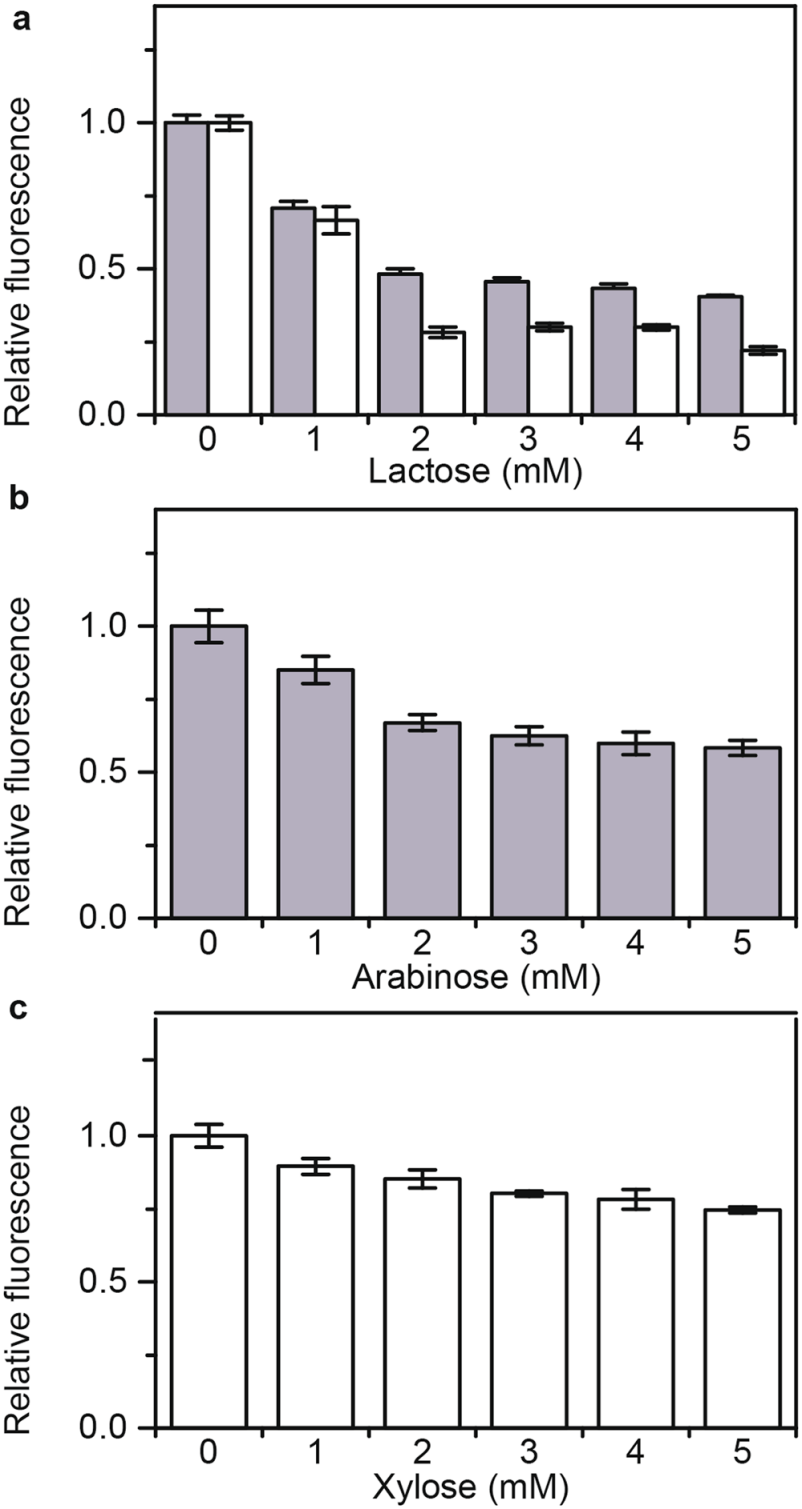
Reciprocal repression of lactose, arabinose, and xylose gene expression. (**a**) Effect of increasing concentrations of lactose on *araB* (grey bars) and *xylA* (white bars) promoter activity as determined using transcriptional fusions to the fluorescent protein Venus (P_*araB*_-Venus and P_*xylA*_-Venus). Cells were grown in TB7 with 1 mM arabinose or xylose. (**b**) Effect of increasing arabinose concentrations on *lacZ* promoter activity as determined using transcriptional fusions to the fluorescent protein Venus (P_*lacZ*_-Venus). Cells were grown in TB7 with 1 mM lactose. (**c**) Effect of increasing xylose concentrations on *lacZ* promoter activity as determined using transcriptional fusions to the fluorescent protein Venus (P_*lacZ*_-Venus). Cells were grown in TB7 with 1 mM lactose. Error bars denote the standard deviation of three experiments performed on separated days.

As a first step towards investigating catabolite repression in sugar mixtures involving lactose and arabinose or lactose and xylose, we measured how these sugars individually affect the expression of each other’s metabolic genes. To measure gene expression, we employed single-copy transcriptional fusions to the fluorescent protein Venus. Briefly, we grew the cells in TB7 medium containing 1 mM of the cognate sugar, which is sufficient to fully activate the expression of the corresponding metabolic genes, and increasing concentrations of a second sugar. As shown in Fig. 1a, lactose inhibited expression from both the *araB* and *xylA* promoters, which respectively control the expression of the arabinose and xylose metabolic genes. These results are consistent with the results reported by Aidelberg and coworkers^14^. In addition, we found that lactose represses the *xylA* promoter more strongly than the *araB* promoter.

We also tested whether arabinose and xylose inhibit the *lacZ* promoter, which controls the expression of the lactose metabolic genes. As shown in Fig. 1b, arabinose represses expression from the *lacZ* promoter. These results indicate that the repression is reciprocal. In other words, lactose represses arabinose metabolism and arabinose represses lactose metabolism. However, repression by lactose is somewhat greater than repression by arabinose. In the case of xylose (Fig. 1c), we found that it weakly represses expression from the *lacZ* promoter. While repression is also reciprocal during growth on lactose and xylose, lactose is clearly the preferred sugar.

We also performed identical experiments in M9 minimal medium^13,18^. Similar to what was observed in TB7, we found that lactose inhibits the expression of the arabinose and xylose metabolic genes in M9 minimal medium (Figure S1), though the effect is stronger in this medium. In addition, we found that arabinose again weakly represses the expression of the lactose metabolic genes. However, xylose was found to have no significant effect on the expression of the lactose genes in M9 minimal medium.

### Lactose reduces the rate of arabinose and xylose consumption

**Figure 2:**
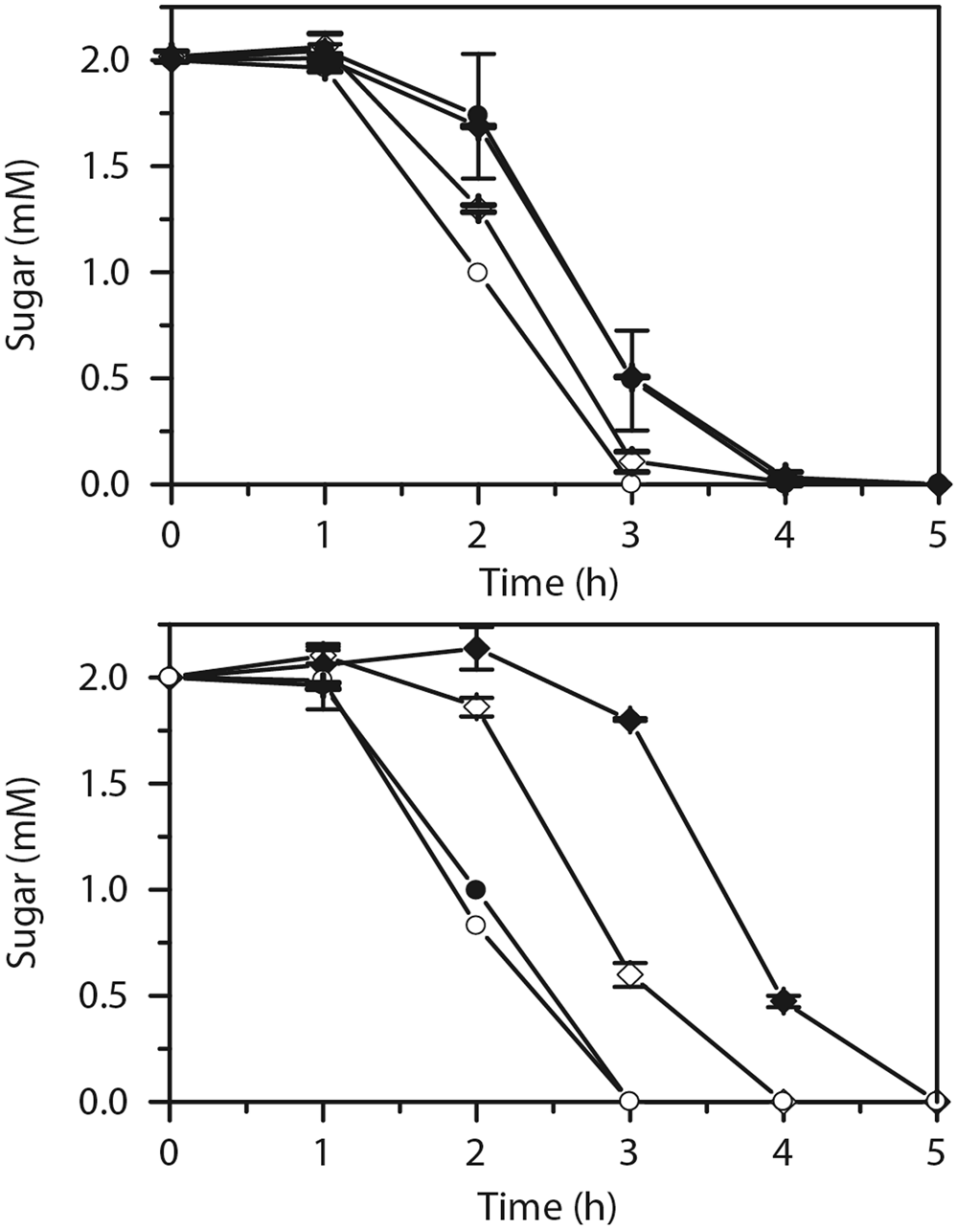
Lactose inhibits the utilization of arabinose and xylose. (**a**) Sugar utilization in mixtures of lactose and arabinose. Diamonds refer to arabinose concentrations in the presence (black) or absence (white) of lactose; circles refer to lactose concentrations in the presence (black) or absence (white) of arabinose. (**b**) Sugar utilization in mixtures of lactose and xylose. Diamonds refer to xylose concentrations in the presence (black) or absence (white) of lactose; circles refer to lactose concentrations in the presence (black) or absence (white) of xylose. Cells were grown in TB7. Error bars denote the standard deviation of three experiments performed on separated days.

We next investigated sugar utilization during growth on arabinose or xylose in the presence of lactose. We grew the cells in sugar mixtures containing equal concentrations on a molar basis of the two sugars (2 mM). As shown in Fig. 2, the cells individually consume lactose and arabinose at roughly the same rates. However, the rate of utilization for both sugars was decreased somewhat when the cells were grown in the presence of both. These results are consistent with a mechanism of reciprocal repression. Only during growth on lactose and xylose was catabolite repression observed. In this case, lactose significantly reduced the rate of xylose consumption. However, xylose did not reduce lactose consumption. Unlike arabinose, xylose is intrinsically consumed more slowly than lactose. Moreover, the rate of xylose consumption is further reduced in the presence of lactose, most likely due to repression of the xylose metabolic genes. The results are consistent with our gene expression results (Fig. 1), where lactose exhibits a greater effect on xylose genes than on arabinose genes.

We also measured the sugar utilization in M9 minimal medium. As shown in Figure S2, lactose delayed the consumption of both arabinose and xylose. These results are consistent with those obtained using TB7 (Fig. 2). However, in M9 minimal medium, arabinose did not delay the utilization of lactose, unlike the case with TB7 medium. In addition, both lactose and xylose were utilized at similar rates when they were individually added to the growth medium. These results demonstrate that lactose represses both the utilization of arabinose and xylose in M9 minimal medium. However, reciprocal repression does not occur in this medium.

### Lactose catabolite repression does not involve transcriptional crosstalk

**Figure 3:**
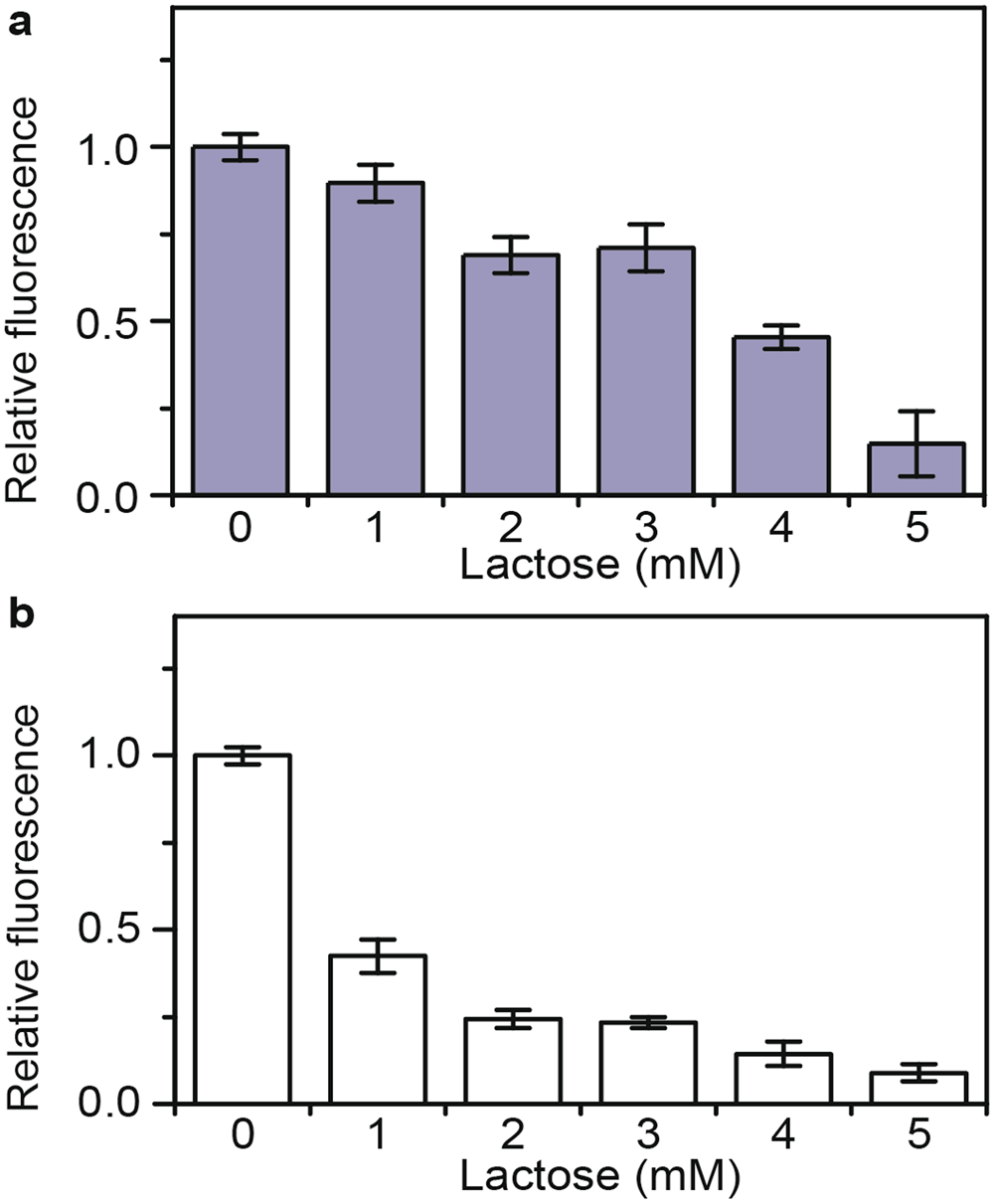
Lactose catabolite repression does not involve transcriptional crosstalk by LacI. (**a**) Effect of increasing concentrations of lactose on *araB* promoter activity in Δ*lacI* mutant (Δ*lacI* P_*araB*_-Venus). Cells were grown in TB7 with 1 mM arabinose. (**b**) Effect of increasing concentrations of lactose on *xylA* promoter activity in Δ*lacI* mutant (Δ*lacI* P_*xylA*_-Venus). Cells were grown in TB7 with 1 mM xylose. Error bars denote the standard deviation of three experiments performed on separated days.

Previous work demonstrated that arabinose represses xylose metabolism and vice versa through a mechanism involving transcriptional crosstalk^12^. In particular, arabinose-bound AraC binds the *xylA* promoter and represses transcription. Similarly, xylose-bound XylR binds the *araB* promoter and represses transcription, albeit to a lesser extent. One possibility is that lactose represses arabinose and xylose gene expression also using competitive mechanism. Briefly, the transcriptional repressor LacI regulates the expression of the lactose genes. When LacI is bound with allolactose, a by-product of lactose metabolism, it no longer binds the *lacZ* promoter and represses transcription. We therefore hypothesized that allolactose-bound LacI binds to the *araB* and *xylA* promoters and represses transcription of the respective metabolic genes. To test this hypothesis, we measured expression from the *araB* and *xylA* promoters in a Δ*lacI* mutant. As shown in Fig. 3, lactose was still able to repress *araB* and *xylA* promoters in the absence of LacI. These results demonstrate that lactose catabolite repression does not involve transcriptional crosstalk by LacI.

### Lactose-mediated repression is independent of arabinose and xylose metabolism

**Figure 4:**
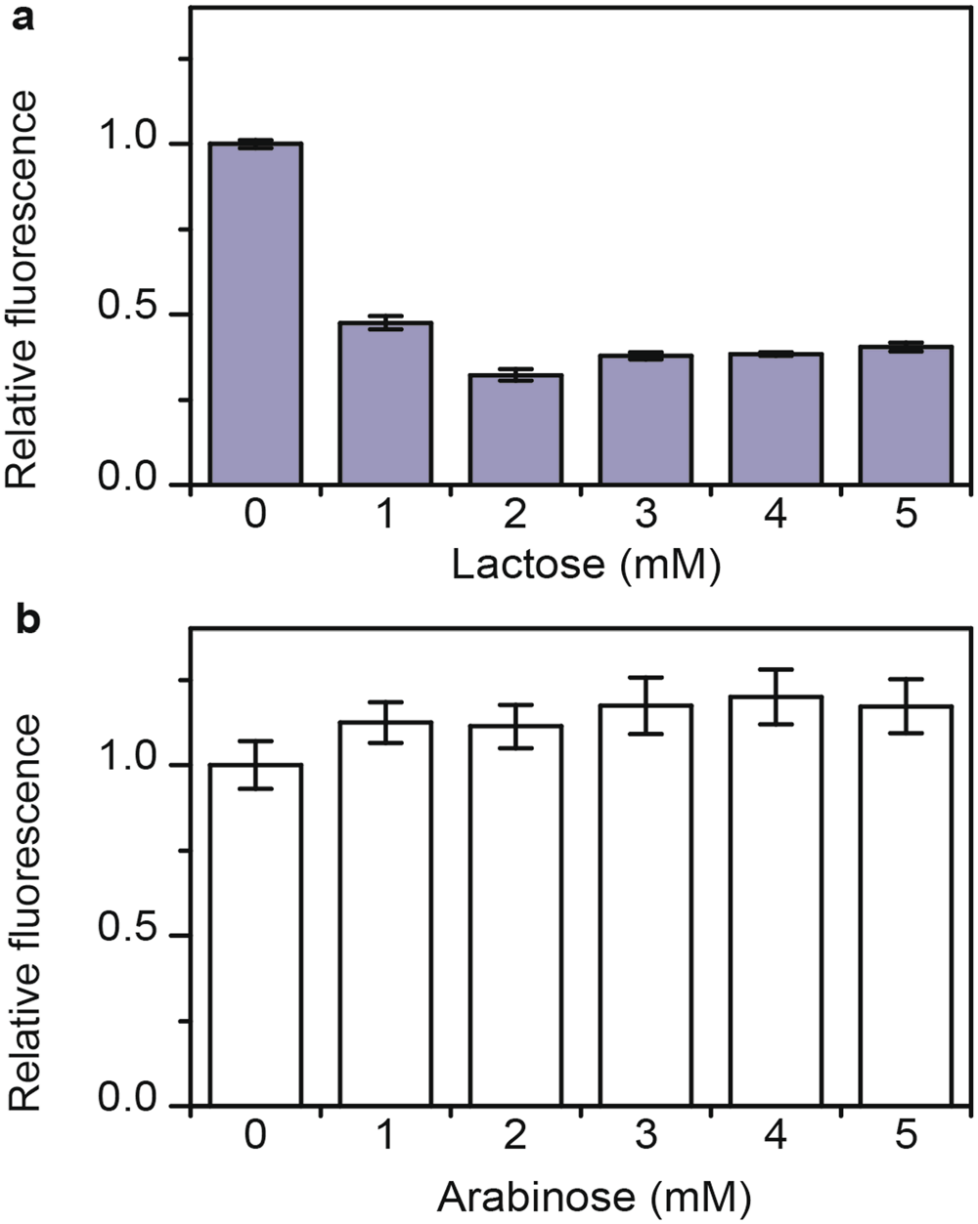
Lactose-mediated repression is independent of arabinose metabolism. (**a**) Effect of increasing concentrations of lactose on *araB* promoter activity in Δ*araBAD* mutant (Δ*araBAD* P_*araB*_-Venus). Cells were grown in TB7 with 1 mM arabinose. (**b**) Effect of increasing concentrations of arabinose on *lacZ* promoter activity in Δ*araBAD* mutant (Δ*araBAD* P_*lacZ*_-Venus). Cells were grown in TB7 with 1 mM lactose. Error bars denote the standard deviation of three experiments performed on separated days.

**Figure 5:**
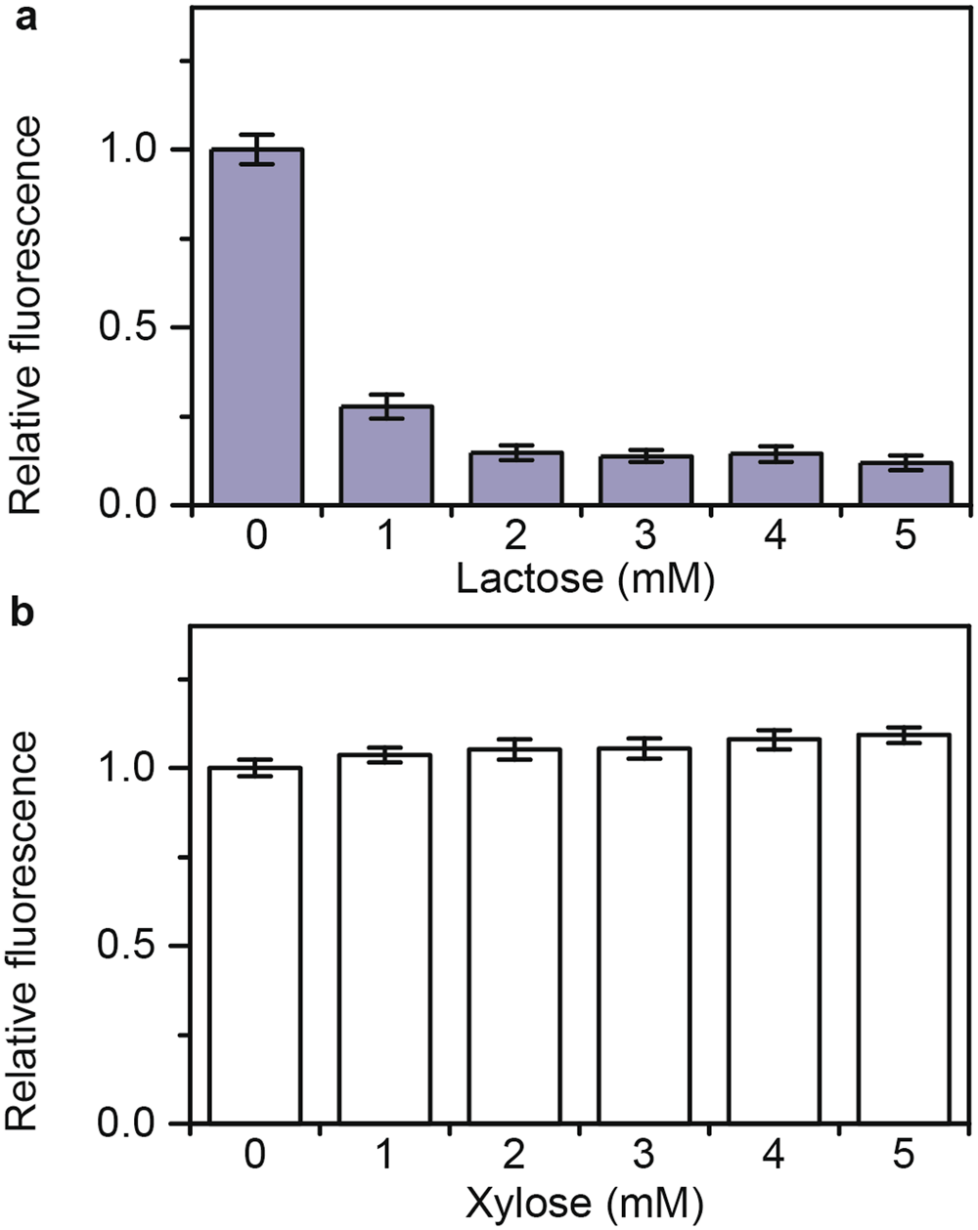
Lactose-mediated repression is independent of xylose metabolism. (**a**) Effect of increasing concentrations of lactose on *xylA* promoter activity in Δ*xylAB* mutant (Δ*xylAB* P_*xylA*_-Venus). Cells were grown in TB7 with 1 mM xylose. (**b**) Effect of increasing concentrations of xylose on *lacZ* promoter activity in Δ*xylAB* mutant (Δ*xylAB* P_*lacZ*_-Venus). Cells were grown in TB7 with 1 mM lactose. Error bars denote the standard deviation of three experiments performed on separated days.

We next tested whether lactose is able to repress arabinose and xylose gene expression in the absence of arabinose and xylose metabolism. The goal here was to see whether lactose was affecting some downstream metabolic process that somewhat would affect arabinose and xylose gene expression. As shown in Figs 4a and 5a, lactose still inhibited arabinose and xylose gene expression in strains unable to metabolize these respective sugars. These results demonstrate that lactose-mediated repression of the arabinose and xylose genes is independent of arabinose and xylose metabolism.

### Arabinose and xylose-mediated repression of the lactose metabolism genes is respectively dependent on arabinose and xylose metabolism

We next tested whether the repression of the lactose genes by arabinose and xylose was respectively dependent on arabinose and xylose metabolism. As shown in Figs 4b and 5b, we observed no repression of lactose gene expression in the absence of arabinose and xylose metabolism. The results demonstrate that arabinose or xylose metabolism is necessary for the repression of the lactose genes. They also suggest that the mechanism for repression involves cAMP. As the respective regulators, AraC and XylR, were still present in these mutants, these results also indicate that the mechanism does not involve transcriptional crosstalk.

We also performed the reciprocal experiment, where we tested whether lactose is able to repress arabinose and xylose gene expression in the absence of lactose metabolism using Δ*lacZ* mutant. Not surprisingly, we observed no repression (data not shown). However, these experiments were uninformative, because they provide no insight regarding the mechanism of lactose-mediated repression. The reason is that LacZ, the enzyme that cleaves lactose into glucose and galactose, also produces allolactose, the inducer for LacI^19^. Thus, we cannot determine whether loss of repression in a Δ*lacZ* mutant results from the loss of the LacI inducer or the inability of the cells to utilize lactose.

### Repression by lactose can be explained by preferential promoter activation by cAMP-CRP

**Figure 6:**
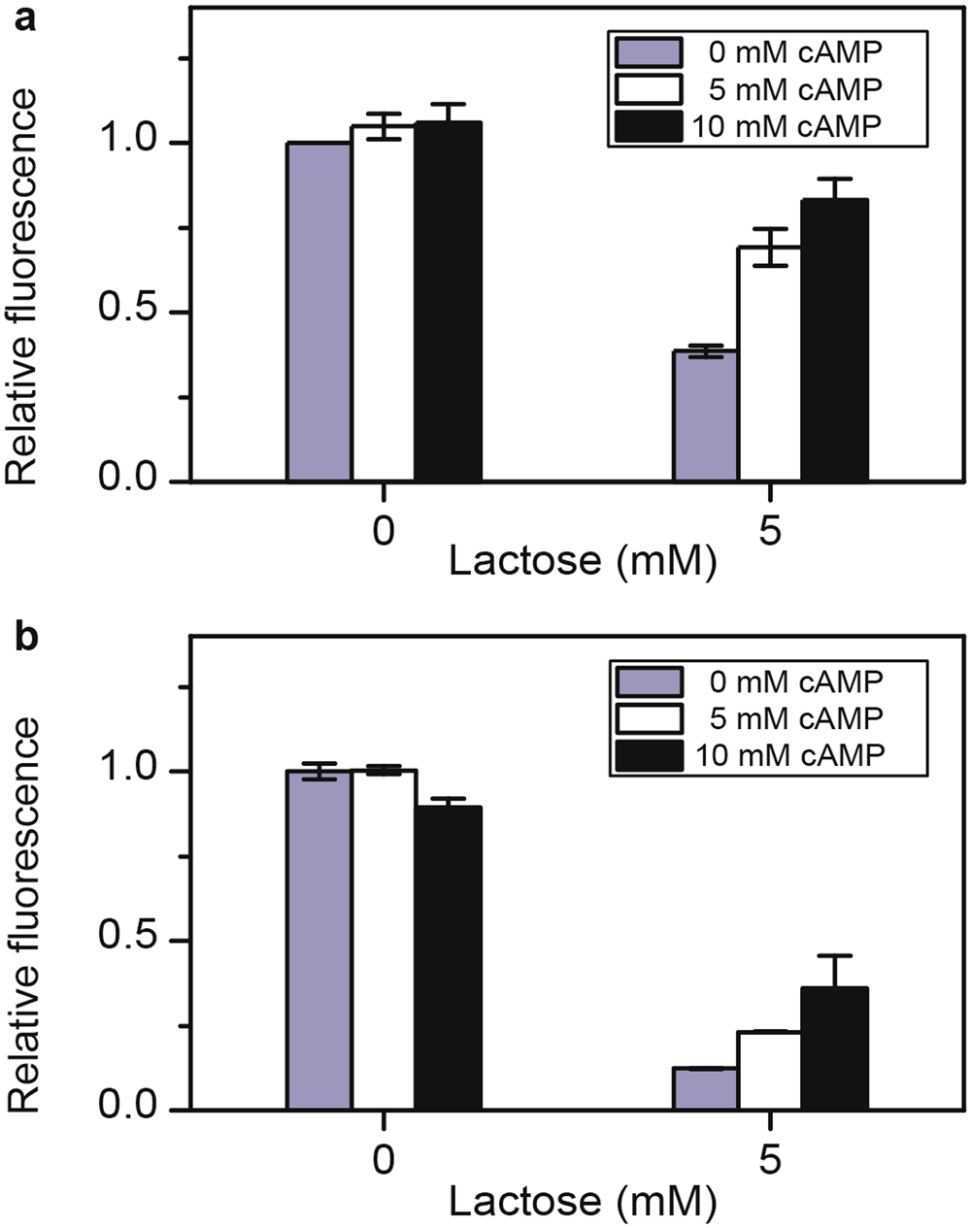
cAMP mitigates lactose-mediated repression of arabinose and xylose gene expression. (**a**) Effect of different concentrations of cAMP on *araB* promoter activity. Cells were grown in M9 minimal medium containing 1 mM arabinose. (**b**) Effect of different concentrations of cAMP on *xylA* promoter activity. Cells were grown in M9 minimal medium containing 1 mM xylose. Error bars denote the standard deviation of three experiments performed on separated days.

Aidelberg and coworkers previously proposed that the hierarchy among lactose, arabinose and xylose is due to differential promoter activation by the CRP global regulator^14^. To further test this hypothesis, we measured whether the addition of cAMP would relieve lactose inhibition of the arabinose and xylose promoters. As shown in Fig. 6, the addition of external cAMP increased *araB* and *xylA* promoter activities in presence of lactose. In the case of the *araB* promoter, repression was relieved by the addition of 5 or 10 mM cAMP. However, in the case of the *xylA* promoter, repression was only slightly relieved, even though similar concentrations of cAMP were employed. We were unable to test higher concentrations of cAMP (>10 mM), because they hindered cell growth. Nonetheless, these results are consistent with a mechanism whereby lactose inhibits arabinose and xylose metabolism through CRP.

We also tested whether the addition of cAMP would relieve the repression of the *lacZ* promoter by arabinose. As shown in Figure S3, we found that repression was partially relieved by the addition of 5 mM cAMP. Higher concentrations of cAMP (10 mM), however, did not further increase the activity of the *lacZ* promoter. These results demonstrate that arabinose-mediated repression of the *lacZ* promoter is due in part to cAMP. Whether it represents the sole mechanism is unknown.

### Swapping the CRP binding site of *xylA* promoter with that of *lacZ* promoter reduces lactose-mediated repression of xylose metabolism

**Figure 7:**
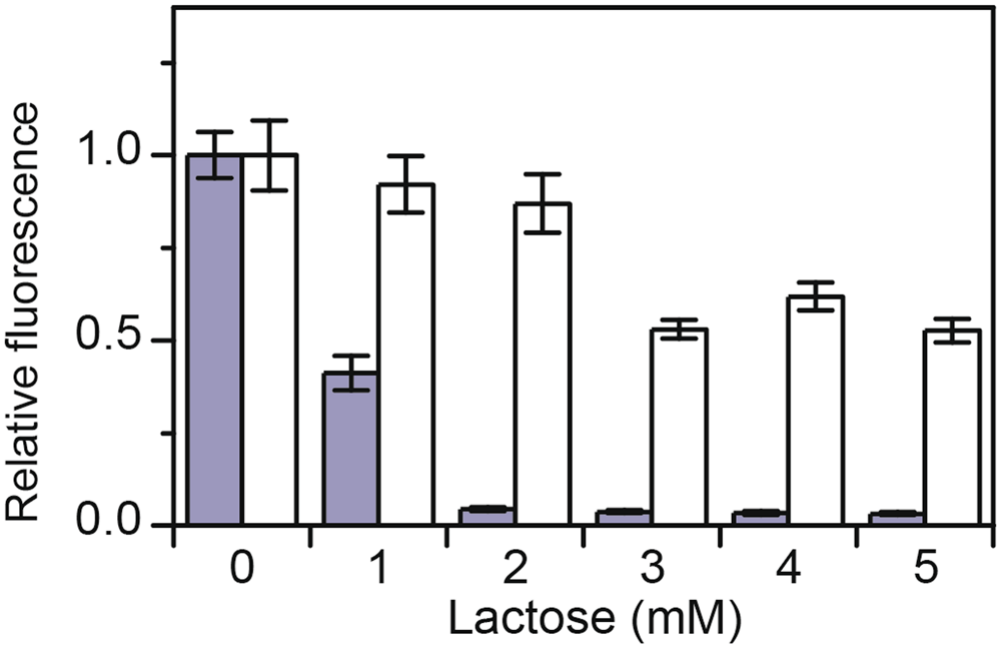
Swapping the CRP binding site of *xylA* promoter with that of *lacZ* promoter reduces lactose-mediated repression of xylose metabolism. Effect of increasing concentrations of lactose on the activity of *xylA* promoter with native (grey bars) or *lacZ* (white bars) CRP binding site. Cells were grown on M9 minimal medium with 1 mM xylose. Error bars denote the standard deviation of three experiments performed on separated days.

Previous results suggest that the CRP binding site within the *lacZ* promoter has a higher affinity for cAMP-CRP than the CRP binding site within the *xylA* promoter^14^. If catabolite repression is due to differential activation by CRP, then replacing the weak CRP binding site within the *xylA* promoter with a stronger one from the *lacZ* promoter should relieve repression. The *xylA* promoter has a single CRP binding site^20^. The *lacZ* promoter has two CRP binding sites^21,22^. The distal site is considered to be the main CRP binding site for the *lacZ* promoter. Therefore, we replaced the native CRP binding site within the *xylA* promoter with the distal CRP binding site from the *lacZ* promoter. As shown in Fig. 7, less repression by lactose was observed with the *xylA* promoter containing the distal *lacZ* CRP binding site than with the one containing the native CRP binding site. These results further support a mechanism where lactose-mediated repression of xylose metabolism is due to cAMP.

## Discussion

*E. coli* is capable of growing on a number of different sugars. While much is known about how *E. coli* regulates its metabolism when grown on a single sugar, far less is known about how this bacterium regulates its metabolism when grown on mixtures of sugars, particularly when the mixture does not involve glucose. A number of studies have shown that *E. coli* will selectively utilize non-glucose sugars, first consuming the sugar that yields the highest growth rate and then consuming the sugar that yields the next highest growth and so on^11,12,14^. Still many questions remain about the mechanisms governing the selective utilization of these sugars. Two mechanisms have recently been proposed. One involves transcriptional crosstalk between the regulatory systems governing the metabolism of individual sugars. This mechanism was shown to govern the preferential utilization of arabinose over xylose^12^. The other mechanism involves cAMP, a second messenger involved in glucose catabolite repression^4,6^. This mechanism was proposed to govern the preferential utilization of multiple non-glucose sugars^14^. However, this study did not consider whether transcriptional crosstalk was also involved. Indeed, cAMP does not appear to govern the preferential utilization of arabinose over xylose^12^. Furthermore, repression is reciprocal in the case of arabinose and xylose, where arabinose strongly represses the expression of the xylose metabolic genes and xylose weakly represses the expression of the arabinose metabolic genes^12^. These results indicate that the hierarchy among sugars is not necessarily strict but rather depends on the relative concentrations of the two sugars.

In this work, we investigated the selective utilization of three sugars in *E. coli*: lactose, arabinose, and xylose. Our goal was to determine whether transcriptional crosstalk or cAMP/CRP governed the selective utilization of these sugars. In rich medium, we found that both lactose and arabinose inhibited the utilization of the other (Fig. 2b). Consistent with these results, we found that lactose inhibits the expression of the arabinose metabolic genes and that arabinose inhibits the expression of the lactose metabolic genes. In minimal medium, however, lactose is the preferred sugar. These results are also consistent with gene expression data (Figure S1), where lactose is a stronger repressor of arabinose gene expression than arabinose is of lactose gene expression. The origin of these differences on rich and minimal media is not known, though may reflect the complex growth patterns observed in peptide-based medias^23,24^. In the case of xylose and lactose, *E. coli* prefers the latter in both complex and minimal media. Even though xylose inhibits the expression of the lactose genes, the effect is relatively weak.

This hierarchy matches the growth rates associated with these sugars^14^. One question is why does *E. coli* grow more rapidly on lactose than on arabinose or xylose. The increased growth rate associated with lactose is likely due to its metabolism requiring less steps: intracellular lactose is degraded by the enzyme LacZ into galactose and glucose, where the latter can directly enter the Embden–Meyerhof–Parnas (glycolytic) pathway. Arabinose and xylose metabolism involves many more steps, as they both enter the glycolytic pathway through the non-oxidative branch of the pentose phosphate pathway. This likely explains why their metabolism is more inefficient, in addition to their lower energetic yield. Therefore, one would expect that lactose inhibits the metabolism of arabinose and xylose, as this would reflect a more efficient allocation of limited metabolic resources.

Regarding the mechanism, our data support one involving cAMP. Indeed, we found that lactose was still able to repress the expression of the arabinose and xylose metabolic genes when LacI, the cognate regulator of the lactose genes^25^, was deleted (Fig. 3). Had the mechanism involved transcriptional crosstalk, we would expect repression to be eliminated in a Δ*lacI* mutant, contrary to what was observed. These results indicate that lactose metabolism alone is sufficient for repressing the expression of the arabinose and xylose genes. Likewise, we found that arabinose and xylose metabolism were necessary for arabinose and xylose to repress lactose gene expression (Figs 4 and 5).

That metabolism is necessary for repression strongly suggests that cAMP is involved. In support of this mechanism, we found that the addition of exogenous cAMP partially relieved lactose-mediated repression of arabinose and xylose gene expression (Fig. 6). In the case of the arabinose genes, exogenous cAMP was nearly able to counteract the repressive effects of lactose. In the case of the xylose genes, however, exogenous cAMP was only able to partially relieve the repression by lactose, possibly due to our inability to explore higher cAMP concentrations^15–17^. As a further test, we also replaced the native CRP binding site within the *xylA* promoter with one from the *lacZ* promoter. Consistent with a mechanism involving cAMP, we found that lactose-mediated repression was reduced when the CRP site was replaced in the *xylA* promoter (Fig. 7).

While our results demonstrate that cAMP regulates the sugar utilization in mixtures of lactose and arabinose or xylose, it may not be the only mechanism involved. One possibility is that allolactose, a byproduct of lactose metabolism and the inducer for LacI^19^, could inhibit AraC and XylR, the respective regulators of arabinose and xylose gene expression^20,26,27^. Previous work has shown that isopropyl β-D-1-thiogalactopryanoside (IPTG), a non-metabolizable lactose analog, competitively inhibits AraC^28^. We tested whether it competitively inhibits XylR. However, we found that it had no effect on xylose gene expression (data not shown). We also tested whether thiomethyl galactoside (TMG), another non-metabolizable lactose analog, inhibits arabinose and xylose gene expression (Figure S4). Consistent with the IPTG results, we found that TMG competitively inhibits arabinose gene expression but not xylose gene expression (Figure S4). These results suggest that allolactose may competitively inhibit AraC, though whether these non-metabolizable analogs truly mimic the effect of allolactose is unknown. Unfortunately, we were unable to directly test allolactose as this sugar is difficult to obtain. Nevertheless, these results indicate that allolactose likely does not repress XylR. Furthermore, as lactose represses xylose gene expression more strongly than arabinose gene expression, it seems unlikely that inhibition of AraC by allolactose, assuming it occurs, is a significant contributor to catabolite repression.

Why does *E. coli* employ different mechanisms for selective sugar utilization? As noted above, the mechanism differs for a mixture involving arabinose and xylose and those involving lactose and arabinose or lactose and xylose. One possibility is that these differences reflect the relative frequency of encountering these different sugar mixtures in nature. In particular, *E. coli* is unlikely to encounter sugar mixtures involving lactose and arabinose or lactose and xylose. Lactose is found in milk whereas arabinose and xylose are found in the complex polysaccharides that comprise the plant cell wall^29^. In other words, cells are unlikely to be exposed to mixture of lactose and arabinose or lactose and xylose whereas they will likely be exposed to mixture of arabinose and xylose. This would suggest that cAMP is a general mechanism for catabolite repression whereas transcriptional crosstalk is a more specialized one. In particular, mechanisms involving transcriptional crosstalk likely evolved to regulate more frequent events than the generalized mechanism involving cAMP, which appears not to be adapted for any specific sugars.

In summary, we investigated the mechanism for catabolite repression in sugar mixtures of lactose, arabinose, and xylose. Building on previous work, we established that cAMP principally regulates the selective utilization of lactose and arabinose; and lactose and xylose. Moreover, we found that repression is reciprocal in the sense that both sugars repress each other’s metabolism, though lactose is clearly the dominant sugar. These results further our understanding of metabolism in *E. coli*. They also demonstrate that multiple mechanisms are involved in regulating the metabolism of sugar mixtures not involving glucose.

## Methods

### Media and growth conditions

Luria-Bertani (LB) medium (10 g/L tryptone, 5 g/L yeast extract, and 10 g/L NaCl) was used for strain construction. All experiments were performed in either buffered tryptone broth (TB7: 10 g/L tryptone and 1 mM MgSO_4_, buffered at pH 7 with 100 mM potassium phosphate) or M9 minimal medium (6.8 g/L Na_2_HPO_4_, 3 g/L KH_2_PO_4_, 1 g/L NH_4_Cl, 0.5 g/L NaCl, 2 mM MgSO_4_, 100 μM CaCl_2_, 0.001% thiamine hydrochloride and 4 g/L glycerol). All experiments were performed at 37 °C. Antibiotics were added as needed at the following concentrations: ampicillin, 100 μg/mL; chloramphenicol, 20 μg/mL; kanamycin, 40 μg/mL.

### Strain and plasmid construction

Table 1 lists all strains and plasmids used in this study. Table S1 lists all oligonucleotides used in strain and plasmid construction. All strains are isogenic derivatives of *E. coli* MG1655. Genes were deleted using the method of Datsenko and Wanner^30^. Prior to removing the antibiotic resistant cassette using the FLP recombinase, P1 transduction was used to move the cassette to a clean parental background. The plasmid pXW211 contains a transcriptional fusion of the *xylA* promoter to the fluorescent protein Venus. To construct this plasmid, *xylA* promoter (genomic region: 3730691–3731015) was PCR amplified using the primers SK435F and XW435R, and then the PCR fragment was cloned into the integrative plasmid pVenus using SalI and EcoRI restriction sites. The plasmid pSK460 contains a transcriptional fusion of the *lacZ* promoter to the fluorescent protein Venus. To construct this plasmid, the *lacZ* promoter (genomic region: 366222–366545) was PCR amplified using the primers SK418F and XW418R, and then the PCR fragment was cloned into the integrative plasmid pVenus using SalI and EcoRI restriction sites. The plasmid pXW238 contains a transcriptional fusion of the *xylA* promoter to the fluorescent protein Venus, where the native CRP binding site of the *xylA* promoter (TTTTGCGAGCGAGCGCACACTT) (genomic region: 3730899–3730878)^20,31^ was replaced with the CRP binding site of the *lacZ* promoter (TAATGTGAGTTAGCTCACTCAT) (genomic region: 366394–366415)^32^. To construct this plasmid, the plasmid pXW211 was PCR amplified using the primers EA061F and EA062R, which contain the CRP binding site from the *lacZ* promoter as 5′ overhangs. The resulting PCR fragment was then phosphorylated using T4 polynucleotide kinase and ligated back into a circular plasmid using T4 DNA ligase. The plasmid was then integrated into λ phage attachment sites using the CRIM integration system^33^. After confirming by PCR that a single integration took place, the integrated plasmid was moved into a clean background using P1 transduction^34,35^.

**Table 1:** Strains and plasmids used in this study.

**Strain**	**Characteristics**	**Source or Reference**
MG1655	*λ rph-1* (wild type)	CGSC, Yale University
SK459	*attλ*::[*kan* P_*araB*_-Venus *oriR6K*]	13
XW215	*attλ*::[*kan* P_*xylA*_-Venus *oriR6K*]	
XW330	*attλ*::[*kan* P_*lacZ*_-Venus *oriR6K*]	
EA037	Δ*lacI*::FRT *attλ*::[*kan* P_*araB*_-Venus *oriR6K*]	
EA039	Δ*lacI*::FRT *attλ*::[*kan* P_*xylA*_-Venus *oriR6K*]	
EA040	Δ*lacZ*::FRT *attλ*::[*kan* P_*araB*_-Venus *oriR6K*]	
EA041	Δ*lacZ*::FRT *attλ*::[*kan* P_*xylA*_-Venus *oriR6K*]	
EA046	Δ*araBAD*::FRT *attλ*::[*kan* P_*araB*_-Venus *oriR6K*]	
EA047	Δ*araBAD*::FRT *attλ*::[*kan* P_*lacZ*_-Venus *oriR6K*]	
EA049	Δ*xylAB*::FRT *attλ*::[*kan* P_*lacZ*_-Venus *oriR6K*]	
EA050	Δ*xylAB*::FRT *attλ*::[*kan* P_*xylA*_-Venus *oriR6K*]	
EA051	*attλ*::[*kan* P_*xylA*_-Venus *oriR6K*] (CRP binding site of *xylA* promoter is swapped with that of *lacZ* promoter)	
**Plasmid**		
pVenus	*kan att* Venus *oriR6K* (CRIM plasmid with Venus reporter)	
pInt-ts	*bla int oriR6K* (helper plasmid for *att λ* integration)	33
pXW211	*kan att* P_*xylA*_-Venus *oriR6K* (CRIM plasmid with P_*xylA*_-Venus reporter)	
pXW238	pXW211 in which (CRP binding site of *xylA* promoter is swapped with that of *lacZ* promoter)	
pSK460	*kan att* P_*lacZ*_-Venus *oriR6K* (CRIM plasmid with P_*lacZ*_-Venus reporter)	

### Fluorescence assays

In the experiments involving TB7 as the base medium, the cultures were first grown overnight in TB7. The overnight culture was then diluted 1:100 into fresh media and grown for 2 h to an OD_600_ of ~0.2. The cultures were then subcultured into 1 mL of fresh TB7 medium containing the specified sugar to an initial OD_600_ of 0.01 in 96 deep-well plates. The cultures were then incubated on a microplate shaker at 600 rpm for 5 h. In the experiments involving M9 as the base media, the cells were grown overnight in M9 minimal media. The overnight culture was diluted 1:50 into fresh media and grown for 5 h to an OD_600_ of ~0.2. One mL aliquots were then distributed to individual wells in a 96 deep-well plate. The specified sugars were then added to each well, and then the plate was incubated on the microplate shaker at 600 rpm for 4 h. At the end of every experiment, 100 µL samples were taken from each well and transferred to a black, clear-bottom Costar 96-well microplate. Fluorescence (excitation at 515 nm; emission at 528 nm) and absorbance (OD_600_) were measured using Tecan Safire2 microplate reader. The fluorescence readings were normalized with the OD_600_ to account for cell density. All experiments were performed on three separate days, and the average values with the standard deviations are reported.

### Analytical measurements

Cells were first grown overnight in the specified media. The overnight culture was then sub-cultured into 60 mL of fresh media containing the specified sugars in a 125 mL shake flask with an initial OD_600_ of 0.05. The flasks were then incubated on a standard orbital shaker at 250 rpm and 37 °C. Samples (1 mL) were taken at specified time intervals and filtered through 0.22 μm filters (Millipore) for subsequent analysis. Arabinose, lactose and xylose concentrations were measured using a Shimadzu HPLC system equipped with Shimadzu RID-10A refractive index detector (Shimadzu Corp., Kyoto, Japan), and an Aminex HPX-87H column (300 mm × 7.8 mm) (Bio-Rad Laboratories, Hercules, CA, USA). The HPLC oven was adjusted to 65 °C and a mobile phase of 0.5 mM H_2_SO_4_ was run at 0.6 mL/min for 15 min per sample. Kinetic experiments were run in duplicates with the averages and standard deviations reported.

### Data availability

Any other datasets generated during and/or analyzed during the current study are available from the corresponding author upon request.

## Electronic supplementary material

Supplementary Information
