# Supplementary material for: Regulation of metabolism in Escherichia coli during growth on mixtures of the non-glucose sugars: arabinose, lactose, and xylose
Source: Sci Rep. 2018 Jan 12;8:609. doi: 10.1038/s41598-017-18704-0 (PMC5766520; doi:10.1038/s41598-017-18704-0)
Supplement: Supplementary file 1 — Supplementary Information [file 41598_2017_18704_MOESM1_ESM.pdf]

Supplementary Information

Regulation of metabolism in *Escherichia coli* during growth on mixtures of the non-glucose sugars: arabinose, lactose, and xylose

Ehab M. Ammar[1,2], Xiaoyi Wang[1] and Christopher V. Rao[1]*

[1]Department of Chemical and Biomolecular Engineering, University of Illinois at Urbana-Champaign, Urbana, IL 61801, USA

[2]Genetic Engineering and Biotechnology Research Institute, University of Sadat City, El-Sadat City, Egypt

*Corresponding author's mailing address: Department of Chemical and Biomolecular Engineering, University of Illinois at Urbana-Champaign, 600 S. Mathews Ave., Urbana, IL 61801, USA.

Phone: (217) 244-2247; Fax: (217) 333-5052; Email: cvrao@illinois.edu

Supplementary information

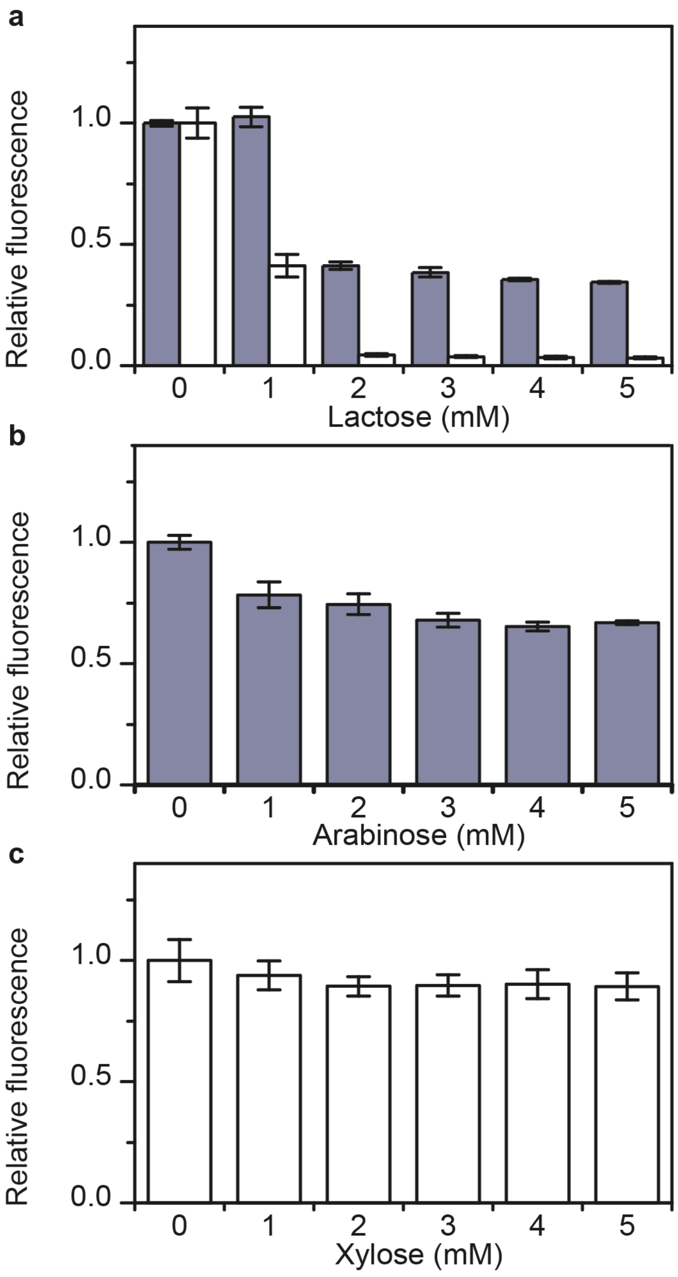

Figure S1. Repression of lactose, arabinose, and xylose gene expression. a. Effect of increasing concentrations of lactose on *araB* (grey bars) and *xylA* (white bars) promoter activity. Cells were grown in M9 minimal medium with 1 mM arabinose or xylose. **b.** Effect of increasing arabinose concentrations on *lacZ* promoter activity. Cells were grown in M9 minimal medium with 1 mM lactose. **c.** Effect of increasing xylose concentrations on *lacZ* promoter activity. Cells

were grown in M9 minimal medium with 1 mM lactose. Error bars denote the standard deviation of three experiments performed on separated days.

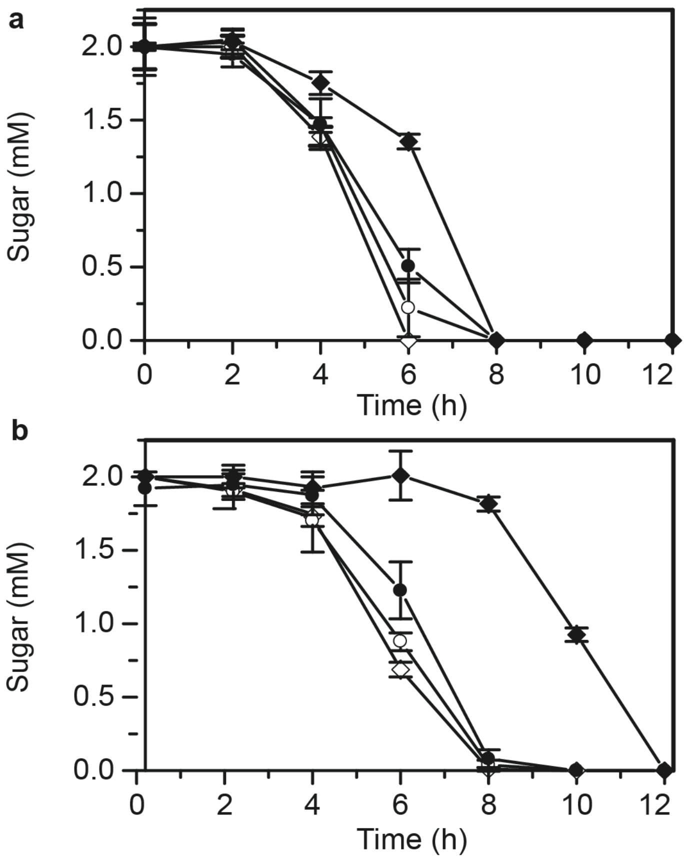

Figure S2. Lactose inhibits the utilization of arabinose and xylose. a. Sugar utilization in mixtures of lactose and arabinose. Diamonds refer to arabinose concentrations in the presence (black) or absence (white) of lactose; circles refer to lactose concentrations in the presence (black) or absence (white) of arabinose. **b.** Sugar utilization in mixtures of lactose and xylose. Diamonds refer to xylose concentrations in the presence (black) or absence (white) of lactose; circles refer to lactose concentrations in the presence (black) or absence (white) of xylose. Cells were grown in M9 minimal medium without glycerol. Error bars denote the standard deviation of three experiments performed on separated days.

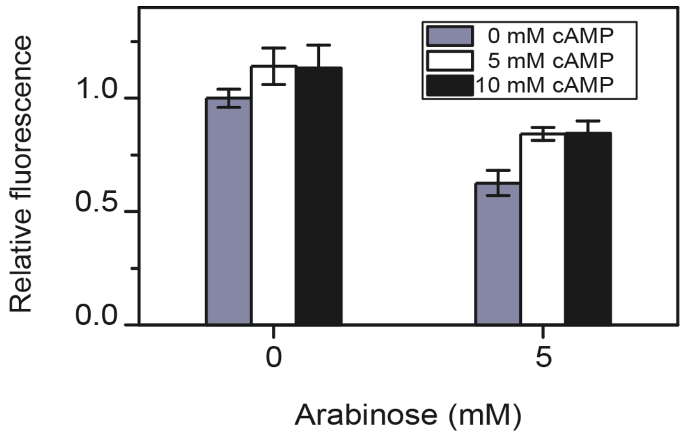

Figure S3. cAMP mitigates arabinose-mediated repression of lactose gene expression. Effect of different concentrations of cAMP on *lacZ* promoter activity. Cells were grown in M9 minimal medium containing 1 mM lactose. Error bars denote the standard deviation of three experiments performed on separated days.

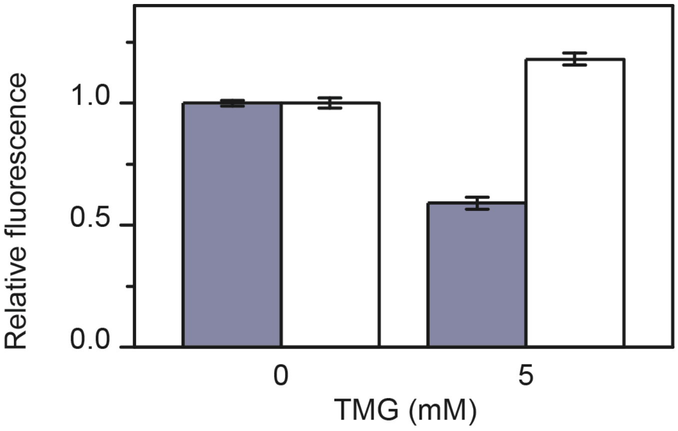

Figure S4. TMG inhibits arabinose gene expression but has no effect on xylose gene expression. Effect of TMG on the activity of *araB* promoter (grey bars) and *xylA* promoter (white bars). Cells were grown in TB7 with 1 mM arabinose or xylose. Error bars denote the standard deviation of three experiments performed on separated days.

Primer	Sequence	Use
SK428F	ATGATAGCGCCCGGAAGAGAGTCAATTCAGG GTGGTGAATGTGTAGGCTGGAGCTGCTTCG	Deleting *lacI*
SK426R	TAGGCCTGATAAGCGCAGCGTATCAGGCAATT TTTATAATCATATGAATATCCTCCTTAG	Deleting *lacI*
EA043F	GGAATTGTGAGCGGATAACAATTTCACACAGG AAACAGCTGTGTAGGCTGGAGCTGCTTC	Deleting *lacZ*
EA044R	ACGCGAAATACGGGCAGACATGGCCTGCCCG GTTATTACATATGAATATCCTCCTTAG	Deleting *lacZ*
TD134F	AGTTTCTTTGATGAGCTGGACCCGATCCTCAA TCGCCATTGTGTAGGCTGGAGCTGCTTC	Deleting *araBAD* [1]
TD134R	TTACTGCCCGTCATATGCCTTCGCGCCATGCT TACGCAGACATATGAATATCCTCCTTAG	Deleting *araBAD* [1]
SK396F	CATCCATCACCCGCGGCATTACCTGATTATGG AGTTCAATGTGTAGGCTGGAGCTGCTTC	Deleting *xylAB* [2]
SK396R	ATGTGAATTATCCCCCACCCGGTCAGGCAGGG GATAACGTCATATGAATATCCTCCTTAG	Deleting *xylAB* [2]
SK435F	AGAGAGGTCGACGATTACGATTTTTGGTTTATT TCTTGATTTATGACCG	PCR amplification of the *xylA* promoter
XW435R	AGAGAGGAATTCACGGAATGCTAACGGGTTTG AG	PCR amplification of the *xylA* promoter
SK418F	AGCTGAGTCGACACTGGTGAAAAGAAAAACCA CC	PCR amplification of the *lacZ* promoter
XW418R	AGCTGAGAATTCAAGGCGATTAAGTTGGGTAA C	PCR amplification of the *lacZ* promoter
EA061F	TAATGTGAGTTAGCTCACTCATGTGAATTATCT CAATAGCAGTGTG	Swapping the CRP binding site of the *xylA* promoter with that from *lacZ* promoter
EA062R	TAAAATGGAATGATGAAACTGGGTAATTCCTC GAAGAG	Swapping the CRP binding site of the *xylA* promoter with that from *lacZ* promoter

Table S1. Primers

1 Desai, T. A. & Rao, C. V. Regulation of arabinose and xylose metabolism in *Escherichia coli*. *Appl Environ Microb* **76**, 1524-1532 (2010).

2 Koirala, S., Wang, X. & Rao, C. V. Reciprocal regulation of L-arabinose and D-xylose metabolism in *Escherichia coli*. *J Bacteriol* **198**, 386-393 (2016).
